# Patient predictors of health-seeking behaviour for persons coughing for more than two weeks in high-burden tuberculosis communities: the case of the Western Cape, South Africa

**DOI:** 10.1186/s12913-019-3992-6

**Published:** 2019-03-13

**Authors:** Carmen Christian, Cobus Burger, Mareli Claassens, Virginia Bond, Ronelle Burger

**Affiliations:** 1 Department of Economics, Stellenbosch University, Matieland, 7602 South Africa; 2 Department of Economics, University of the Western Cape, Bellville, 7535 South Africa; 3 Desmond Tutu TB Centre, Department of Paediatrics and Child Health, Stellenbosch University, Tygerberg, 7505 South Africa; 4 Department of Global Health and Development, Faculty of Public Health and Policy, London School of Hygiene and Tropical Medicine, 15-17 Tavistock Place, London, WC1H9SH UK; 5 Zambart, School of Public Health, University of Zambia, PO Box 50697, Lusaka, Zambia

**Keywords:** Presumptive TB, Chronic cough, Delayed health seeking behaviour, Consulting

## Abstract

**Background:**

This study aimed to analyse the patient predictors of health-seeking behaviour for persons coughing for more than 2 weeks to better understand this vulnerable and important population.

**Methods:**

The study analysed data from a cohort study (SOCS - Secondary Outcome Cohort Study) embedded in a community randomised trial ZAMSTAR (Zambia and South Africa TB and AIDS Reduction Study) in eight high-burden TB communities in the Western Cape, South Africa. These datasets are unique as they contain TB-related data as well as data on health, health-seeking behaviour, lifestyle choices, employment, socio-economic status, education and stigma. We use uni- and multivariate logistic regressions to estimate the odds ratios of consulting for a cough (of more than 2 weeks duration) for a range of relevant patient predictors.

**Results:**

Three hundred and forty persons consulted someone about their cough and this represents 37% of the 922 participants who reported coughing for more than 2 weeks**.** In the multivariate analysis, respondents of black ethnic origin (OR 1.99, 95% CI 1.28–3.12, *P* < 0.01), those with higher levels of education (OR 1.05 per year of education, 95% CI 1.00–1.10, *P =* 0.05), and older respondents (OR 1.02 per year, 95% CI 1.01–1.04, *P <* 0.01) had a higher likelihood of consulting for their chronic cough. Individuals who smoked (OR 0.63, 95% CI 0.45–0.88, *P <* 0.01) and those with higher levels of socio-economic status (OR 0.81, 95% CI 0.71–0.92, *P <* 0.01) were less likely to consult. We find no evidence of stigma playing a role in health-seeking decisions, but caution that this may be due to the difficulty of accurately and reliably capturing stigma due to, amongst other factors, social desirability bias.

**Conclusions:**

The low levels of consultation for a cough of more than 2 weeks suggest that there are opportunities to improve case-finding. These findings on health-seeking behaviour can assist policymakers in designing TB screening and active case-finding interventions that are targeted to the characteristics of those with a chronic cough who do not seek care.

**Electronic supplementary material:**

The online version of this article (10.1186/s12913-019-3992-6) contains supplementary material, which is available to authorized users.

## Background

TB transmission is amplified in the context of the prevailing HIV pandemic in the WHO (World Health Organization) African region, where 31% of all new TB cases in adults are attributable to HIV infection [1]. In this context where transmissions occur more readily, delays to seeking healthcare have a higher mortality and morbidity cost [2, 3]. This translates into high health and socio-economic costs (due to loss of or decreased employment), which inevitably places a higher burden on scarce public resources [4, 5].

Delays in seeking healthcare for a cough may arise from the patient or the healthcare system, or, most likely, both. The focus of this study is on understanding how individual characteristics are correlated with the health-seeking decisions of symptomatic cases.

Very few studies comprehensively investigate health-seeking behaviours from the patient perspective exclusively [6, 7]. More often, studies focus on delays in diagnosis to explore both demand and supply dynamics. However, often such approaches do not allow for a detailed exploration of patient considerations.

The most recent systematic review of delays in TB diagnosis found that evidence describing the potential risk factors for delay are heterogeneous [8]. A risk factor in one setting may cause an increased delay while in another setting, a decreased delay. For example, a Zambian study found that HIV has a positive association with the risk of diagnostic delay [9], while studies in Ghana, Spain and Thailand showed a negative association for the same risk factor [10–12]. In addition, some predictors were identified extensively in many studies (namely older age, poverty, low levels of education and lack of TB awareness) while others may only appear in one study (for example, stigma). Based on the systematic review, the following prominent patient correlates were identified, amongst others: health status, socio-economic status, stigma, age, gender, education, TB knowledge levels, smoking and drinking.

Responding to the global call to address stigma in the fight against TB [13, 14], this study is (as far as we know) the first to include a quantitative measure of TB stigma in modelling health-seeking behaviour. Our uniquely comprehensive data set allows us to address this gap in patient-focused analysis of health-seeking decisions. Such findings information can assist policymakers in designing more targeted TB screening and active case-finding interventions.

## Method

### Study design and setting

This cross-sectional study uses SOCS and other ZAMSTAR data to estimate the odds ratios of consulting for a cough (of more than 2 weeks) for relevant patient predictors.

ZAMSTAR was a community-randomised trial that aimed to reduce TB and AIDS in South Africa and Zambia, two countries with amongst the highest TB incidence rates globally (in 2010 TB incidence in Zambia was 495/100000 people and 948/100000 for South Africa [15]), using multiple interventions [16]. Study outcomes were measured in 2010 using prevalence surveys in communities that had TB notification rates of more than 400/100000. In addition to objective health outcomes, the prevalence surveys collected individual-level data on health-seeking behaviour (Question Q41_CAC in ZAMSTAR prevalence survey reads: ‘Did you consult anybody for this cough?), socio-economic status, demographics, self-reported health status and lifestyle choices. This secondary analysis study used data from the eight ZAMSTAR communities in the Western Cape, South Africa. Respondents were aged 18 years and older.

The aim of the SOCS was to recruit TB-affected households in each of the ZAMSTAR communities in order to collect stigma-related data from confirmed TB patients and their family members [17]. This study used the TB-stigma data derived from the first and second round of the SOCS (2008 to 2009) since it was the closest in time to the ZAMSTAR prevalence survey data (2010) and therefore captured the most recent degree of TB-stigma.

These datasets – ZAMSTAR and SOCS - are unique given that they contain TB-related data as well as data on health, health-seeking behaviour, lifestyle choices, employment, socio-economic status, education and stigma. There is currently no other TB-related dataset of this size (*n* = 30,017) in South Africa that provides such a comprehensive range of variables at an individual, household and community level.

### Sample of interest

The sample of interest was restricted to individuals who reported coughing for more than 2 weeks. According to TB-screening guidelines all persons who report coughing for more than 2 weeks should be screened for TB [18].

### Variables of interest

The outcome variable used to capture health-seeking behaviour was a consulting dichotomised variable.

Age, education and an asset index (SES proxy) were included as continuous covariates.

The asset index was derived from household asset ownership indicators using multiple correspondence analysis (MCA) (see *Multiple Correspondence Analysis and Related Methods* [19] for a detailed discussion of the methodology of MCA) to derive the weights. This multidimensional approach to measuring SES is based on seminal work in the development economics literature [20] and is increasingly used in welfare analysis and poverty targeting studies [21]. The initial validation study of the asset index (using data from developing countries Indonesia, Nepal and Pakistan) [20] produced internally coherent results which showed distinct separations across different SES households (poor, middle and rich) for each asset. The index was also robust to the assets included and was comparable with output and poverty levels across countries.

The asset index was composed of the following SES domains: semi-durable assets (television, refrigeration, motor vehicle, mobile phone), access to electricity, dwelling type (including formal and informal structures), domestic assistance, main type of toilet (including flush toilet and bucket system), main source of drinking water (including piped sources and wells), fuel access for keeping warm (including electricity and wood) and reliance on food relief (Additional file 1).

When regressing the components of the asset index on the asset index, the signs and size of the coefficients were as expected given that ownership of or access to more (or superior) assets indicate a higher SES (Additional file 2).

The model also contained dummy variables for gender, employment status, and ethnicity (only two ethnicities were observed in the sample of interest: Individuals of Cape coloured ethnic origin and individuals of black ethnic origin). A proxy for HIV was included as a dichotomous health-status covariate.

Lifestyle-choice binary variables related to smoking and drinking (alcohol) were included. These covariates were the only lifestyle-choice variables available in the ZAMSTAR dataset.

Respondents of the SOCS survey were presented with questions or statements linked to seven TB-stigma domains: unnecessary fears of transmission, blame, experience of social exclusion, experience of being made fun of, experience of health-setting stigma, internal stigma and disclosure (Additional file 3). The first two TB-stigma domains pertain to household members of the confirmed TB patient; the remaining five pertain to the confirmed TB patient.

Only six of the seven stigma domains were used to create the stigma index: We regarded the disclosure of one’s TB status as an outcome rather than a feature of stigma. This presented an endogeneity challenge and therefore we omitted the disclosure domain from our stigma index.

After summing the selected six stigma dummies, the composite variable was transformed into an index using the weighted arithmetic mean. For ease of interpretation, this stigma index was standardised using the z-score.

We calculated a mean stigma index per community, gender and age (Additional file 4) (in the SOCS dataset). These mean values were used to create a stigma index for each ZAMSTAR respondent weighted according to their gender, age and the community in which they lived.

### Data analysis and processing

Descriptive statistics of the covariates were conducted for the sample of interest, as well as the subgroup that consulted and the subgroup that did not. A two-sample t test with equal variances (for continuous covariates) and a chi-square test (for dichotomous covariates) were used to test for significant differences between the two sub-groups.

A logistic regression was used for uni- and multivariate analyses. In the model, the dependent variable captures health-seeking behaviour and relevant independent variables include socio-economic status, demographic, health status, lifestyle choice and stigma.

In line with best practice [22], a regression-error specification test (RESET) was conducted. The model passed the test and is well-specified since the RESET result was insignificant (chi-squared = 1.82; *P =* 0.18).

Empirical analyses were performed using STATA (version 14.02, StataCorp LLC, College Station, TX, USA).

## Results

### Summary of health-seeking outcomes

Three per cent of the full ZAMSTAR sample (922 out of 30,017 persons) reported coughing for more than 2 weeks. These 922 observations constitute the sample of interest. Thirty-seven per cent (340 persons) of this sample consulted someone about their cough, while the majority - 63% (582 persons) - did not.

### Socio-demographic characteristics of persons coughing for more than 2 weeks

The summary statistics of the sample characteristics are described below (Table 1).

**Table 1:** Summary statistics of sample characteristics, 2010

Continuous covariates	Sample mean (*n* = 922)^a^	Std. Dev.	‘Consult’ mean (*n* = 340)	Std. Dev.	‘Did not consult’ mean (*n* = 582)^b^	Std. Dev.
Stigma Index^*^	−0.06	0.88	−0.12	0.90	−0.03	0.87
Age	40.25	14.82	42.29	14.84	39.05	14.68
Education^c^	7.64	3.63	7.66	3.68	7.63	3.60
Asset Index	−0.08	1.07	−0.19	1.15	−0.02	1.02
Dichotomous covariates	Sample proportions (*n* = 922)	Std. Dev.	‘Consult’ proportions (*n* = 340)	Std. Dev.	‘Did not consult’ proportions (*n* = 582)	Std. Dev.
Men^**^	0.42	0.50	0.39	0.49	0.45	0.50
Black ethnicity^***^	0.84	0.37	0.91	0.29	0.80	0.40
Employed	0.29	0.45	0.26	0.44	0.30	0.46
HIV positive^*^	0.19	0.39	0.22	0.42	0.17	0.38
Drinks daily	0.06	0.24	0.07	0.25	0.06	0.24
Smoked^***^	0.46	0.50	0.36	0.48	0.52	0.50

*** *p* < 0.01, ** *p* < 0.05, * *p* < 0.1 (derived from two-sample t test with equal variances (continuous covariates) and chi-square test (dichotomous covariates))

^a^*n* = 921 for age variable

^b^*n* = 581 for age variable

^c^Education = years of education

Source: Own calculations, ZAMSTAR (2010)

The mean age of those who consulted for their 2-week cough was slightly higher; 42 years compared to those who did not consult (39 years). The mean highest level of education for those who consulted and those who did not was the same – namely Grade 8 (8 years of education).

**Fig. 1:**
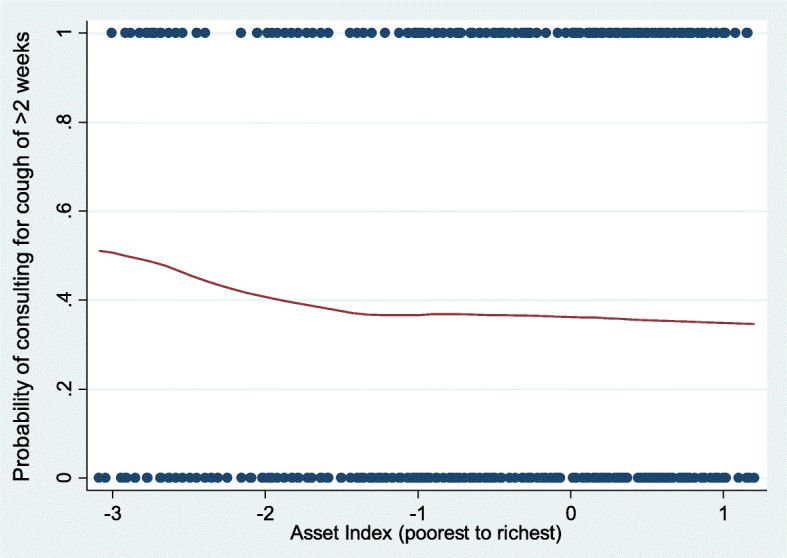
Probability of consulting by SES, 2010. Scatter plot and curve of best fit showing relationship between the probability of coughing (for more than 2 weeks) and the asset index. Source: Own calculations, ZAMSTAR (2010)

The asset index ranged from − 3.08 to 1.20 with a larger index representing a higher level of SES. The negative relationship between the probability of consulting and the asset index (Fig. 1) means that individuals with higher levels of SES were less likely to consult.

Men were significantly less likely to consult than women, with 39% of those consulting being men. The employed were less likely to consult than the unemployed, while individuals of black ethnic origin were significantly more likely to consult than individuals of Cape coloured ethnic origin.

### Clinical and lifestyle characteristics of persons coughing for more than 2 weeks

Twenty-two per cent of those who consulted were HIV positive, while 17% of those who did not consult were HIV positive (*P* = 0.063).

Daily drinking ranged between six and 7 %, with no significant differences between those who consulted and those who did not consult. The proportion of respondents who had ever smoked was significantly higher amongst those who did not consult (52%) than amongst those who did consult (36%).

### Stigma characteristics of persons coughing for more than 2 weeks

The stigma index ranged from − 1.73 (minimum) to 1.78 (maximum) with a larger index representing a higher level of stigma. For those who consulted, the mean stigma level was significantly lower (− 0.12) compared to those who did not (− 0.03).

### Predictors of health-seeking behaviour

Results for the univariate and multivariate logistic regressions (Table 2) are reported in brackets as follows: ORs, 95% CIs and *P-*values for the univariate regression are described first, followed by the results for the multivariate regression.

**Table 2:** Univariate and multivariate logistic regression analysis for consulting for a cough (when coughing for more than 2 weeks), 2010

Variables	Univariate (*n* = 922)^a^		Multivariate (*n* = 921)^b^	
	OR^c^ (95% CI^d^)	*P* value	OR (95% CI)	*P* value
Stigma index	0.88 (0.76–1.03)	0.13	1.05 (0.89–1.24)	0.58
Age^e^	1.01 (1.01–1.02)	< 0.01	1.02 (1.01–1.04)	< 0.01
Men	0.76 (0.57–0.99)	0.04	0.88 (0.63–1.22)	0.44
Black ethnicity	2.42 (1.60–3.67)	< 0.01	1.99 (1.28–3.12)	< 0.01
Education^f^	1.00 (0.97–1.04)	0.87	1.05 (1.00–1.10)	0.05
Employed	0.85 (0.63–1.15)	0.29	0.92 (0.67–1.27)	0.63
Asset index	0.87 (0.77–0.98)	0.03	0.81 (0.71–0.92)	< 0.01
HIV positive	1.37 (0.98–1.91)	0.06	1.30 (0.91–1.85)	0.15
Drinks daily	1.13 (0.66–1.95)	0.65	1.43 (0.80–2.54)	0.23
Smoked	0.53 (0.40–0.69)	< 0.01	0.63 (0.45–0.88)	< 0.01

^a^*n* = 921 for age variable

^b^ Due to one missing age variable

^c^ OR = odds ratio

^d^CI = confidence interval

^e^ per year

^f^per year of education

Reference categories for dichotomous variables: Women, Cape coloured ethnicity, Not employed during last year, HIV negative/did not disclose HIV status, Never/occasional/used to drink, Never smoked. All other independent variables are continuous. Source: Own calculations, ZAMSTAR (2010)

*Stigma* has a statistically insignificant relationship with the probability of seeking healthcare for a cough of more than 2 weeks (OR 0.88, 95% CI 0.76–1.03, *P =* 0.13; OR 1.05, 95% CI 0.89–1.24, *P =* 0.58). The stigma result remained insignificant (OR 0.96, 95% CI 0.82–1.12, *P* = 0.59) even when the multivariate regression was rerun without the variables for SES and ethnicity.

The odds ratio of *men* consulting is less than one in the univariate regression (OR 0.76, 95% CI 0.57–0.99, *P =* 0.04) but this becomes statistically insignificant in the multivariate regression (OR 0.88, 95% CI 0.63–1.22, *P =* 0.44)*. Employment* does not show any significant relationship with the likelihood of consulting though the likelihood of the employed consulting is consistently less than the unemployed (OR 0.85, 95% CI 0.63–1.15, *P =* 0.29; OR 0.92, 95% CI 0.67–1.27, *P =* 0.63). Being of *black ethnic origin* and a year *older* is associated with a higher likelihood of consulting (OR 2.42, 95% CI 1.60–3.67, *P* < 0.01; OR 1.99, 95% CI 1.28–3.12, *P* < 0.01 and OR 1.01, 95% CI 1.01–1.02, *P <* 0.01; OR 1.02, 95%CI 1.01–1.04, *P <* 0.01, respectively). In the case of respondents of black ethnic origin, the reference case is respondents of Cape coloured ethnic origin.

The odds ratio of consulting for each standard deviation increase in the *asset index* was less than one (OR 0.87, 95% CI 0.77–0.98, *P =* 0.03; OR 0.81, 95% CI 0.71–0.92, *P <* 0.01). For each additional year of *education,* the odds of consulting was 1.05 times more likely in the multivariate results (OR 1.00, 95% CI 0.97–1.04, *P =* 0.87; OR 1.05, 95% CI 1.00–1.10, *P =* 0.05).

*HIV-positive* individuals were more likely to consult than their HIV-negative counterparts or those who did not disclose their HIV status (OR 1.37, 95% CI 0.98–1.91, *P =* 0.06; OR 1.30, 95% CI 0.91–1.85, *P =* 0.15) but these findings are not statistically significant in the multivariate regression. There was no significant increased likelihood of consulting for *drinking daily* (OR 1.13, 95% CI 0.66–1.95, *P =* 0.65; OR 1.43, 95% CI 0.80–2.54, *P =* 0.23). Persons *having smoked* were significantly less likely to consult (OR 0.53, 95% CI 0.40–0.69, *P <* 0.01; OR 0.63, 95% CI 0.45–0.88, *P <* 0.01).

## Discussion

Only 37 % of respondents had consulted someone when coughing for more than 2 weeks. Respondents of black ethnic origin (OR 1.99, 95% CI 1.28–3.12, *P* < 0.01), those with higher levels of education (OR 1.05 per year of education, 95% CI 1.00–1.10, *P* = 0.05), and older respondents (OR 1.02 per year, 95% CI 1.01–1.04, *P* < 0.01) had a higher likelihood of consulting for a chronic cough. The opposite held for those who smoked (OR 0.63, 95% CI 0.45–0.88, *P* < 0.01) and those with higher levels of socio-economic status (OR 0.81, 95% CI 0.71–0.92, *P* < 0.01).

### Limitations

The nature of cross-sectional analysis restricts the scope for proving causality. No data were available for one of the more prominent predictors of health-seeking behaviour in the TB literature: TB knowledge levels.

Our data is derived from communities where there is a high prevalence of TB. It is plausible that a gap would exist between stigma scores of TB positive households and non-TB positive households in these communities. However, most important is the relative stigma scores and that it is comparable across communities.

### Interpretation

Compared to similar studies in developing countries, the proportion of respondents who consulted for a chronic cough is relatively low [23, 24]. This is a worrying finding considering that data were collected in high burden communities with TB notification rates of more than 400/100000.

Using the multivariate analysis approach we find no evidence of a role for stigma in health-seeking behaviour amongst persons coughing for more than 2 weeks, but caution against assuming that this means stigma does not present an important constraint to health-seeking behaviour. Given the strong qualitative evidence of a role for stigma in health-seeking [25], one explanation for the lack of quantitative evidence may be that it is not measured well given the challenges of truthfully extracting information on prejudice and bias against TB sufferers. The insignificance of our stigma variable alongside others may thus be partly attributable to the difficulty in accurately capturing stigma via self-reported measures due to social desirability bias and partly to not triangulating this finding with other data sources, including qualitative data. These findings add impetus to the global call for improved tools for measuring stigma and evaluating stigma interventions [26].

Contrary to some studies on health-seeking behaviour [3, 27–29], gender and employment status do not seem to be significantly associated with the probability of seeking healthcare in the multivariate analysis. However, the models consistently show that men are less likely to consult than women, which is in keeping [8, 24, 30–33] with health-seeking literature.

The employed and those with higher levels of SES are consistently less likely to consult. This negative association is supported by most SES and health-seeking findings [6, 34–36]. It is conceivable that below a certain household income threshold, the opportunity cost of working would be relatively higher when compared to those above the threshold. This may explain the negative relationship between these SES variables and health-seeking behaviour.

Cultural dynamics not fully captured in the stigma variable may explain the relatively large, statistically significant differential in the relationship between ethnicity and health-seeking behaviour. Confirming previous findings in the health-seeking literature [8], we find that the educated were significantly more likely to consult. Older individuals were also more likely to consult. We accounted for the possibility of a non-linear relationship between age and health-seeking behaviour in the multivariate analysis (OR 1.00, *P =* 0.17) and found that at older ages this relationship persists, which is in contrast with the health-seeking literature [7, 8].

The positive association between HIV and consulting is not significant, but this may be due to an under-reporting of HIV positive cases.

Having smoked is the only significant lifestyle choice variable, with those having smoked being less likely to consult. It is possible that those who have smoked attribute their symptoms to smoking rather than TB [37]. This relationship may be driven by the harmful consequences smoking has on the lungs and immune system. It is also plausible that having ‘ever smoked’ captures unobservable characteristics which may influence one’s likelihood of consulting.

### Generalisability

External validity is limited to high TB-prevalence communities in South Africa but given South Africa’s high TB prevalence and incidence, these findings are still of international interest. Finding no role for stigma in health-seeking behaviour is important because it highlights the need to triangulate methods and debate how stigma measures interact with other factors in multivariate analyses.

## Conclusion

Most persons coughing for more than 2 weeks did not seek consultation about their chronic cough. Respondents of black ethnic origin, those with higher levels of education and older respondents had a greater likelihood of consulting. Smokers and those with higher SES were less likely to consult.

TB-control programmes should be more cognisant of the patient characteristics of presumptive TB patients who do not seek care. This information can assist policymakers in designing more targeted TB screening and active case-finding interventions. In the high TB burden communities of South Africa, such interventions may benefit from focusing on youth, those with lower levels of education, smokers, higher SES subgroups and also the population of Cape coloured ethnic origin. These findings add to the meagre body of literature analysing the health-seeking behaviour of this vulnerable and important population.

Our study responds to the global call to address stigma in the fight against TB by being the first (as far as we know) to include a quantitative measure of TB stigma in modelling health-seeking behaviour. Our uniquely comprehensive data set allows us to address this gap in patient-focused analysis of health-seeking decisions. Although we find no role for stigma in health-seeking behaviour, it is important because it highlights the need to triangulate methods and debate how stigma measures interact with other factors.

## Additional files

Additional file 1: SES indicator set. Table showing SES components collected in the ZAMSTAR study that was used to create an asset index for this study. (DOCX 13 kb)

Additional file 2: Correlation between components of asset index and asset index. Table showing correlation between components of asset index and asset index. (DOCX 14 kb)

Additional file 3: TB-stigma indicator set. Table showing TB-stigma indicator set used in the SOCS study. (DOCX 13 kb)

Additional file 4: Stigma level by community (a), age (b) and gender (c). Figures (a-c) showing mean stigma index levels (standardised using z-score) for each community (1–8), for each age category (< 18 years, 18–24 years, 25–45 years, 46–60 years, 61+ years) and for each gender (women and men). Source: own calculations, SOCS of ZAMSTAR (2008/2009). (DOCX 26 kb)

## Abbreviations

HIV: Human immunodeficiency virus
MCA: Multiple correspondence analysis
RESET: Regression-error specification test
SES: Socio-economic status
SOCS: Secondary outcome cohort study
TB: Tuberculosis
WHO: World Health Organization
ZAMSTAR: Zambia and South Africa TB and AIDS Reduction
