# Supplementary material for: Patient predictors of health-seeking behaviour for persons coughing for more than two weeks in high-burden tuberculosis communities: the case of the Western Cape, South Africa
Source: BMC Health Serv Res. 2019 Mar 13;19:160. doi: 10.1186/s12913-019-3992-6 (PMC6417175; doi:10.1186/s12913-019-3992-6)
Supplement: Supplementary file 1 — SES indicator set. Table showing SES components collected in the ZAMSTAR study that was used to create an asset index for this study. (DOCX 13 kb) [file 12913_2019_3992_MOESM1_ESM.docx]

**SES indicator set**

| **Domain** | **Items** | **Indicator coding** |
| --- | --- | --- |
| Semi-durable household assets | Television | 1 = yes  0 = no |
|  | Refrigeration | 1 = yes  0 = no |
|  | Motor vehicle | 1 = yes  0 = no |
|  | Mobile phone | 1 = yes  0 = no |
| Household access to electricity | Electricity in the household | 1 = yes  0 = no |
| Dwelling type | 1. House (single unit)  2. House (multi-unit)  3. Traditional dwelling  4. Flat  5. Room in backyard  6. Informal dwelling in backyard  7. Informal dwelling not in backyard  8. Caravan  9. Worker hostel  10. Other (incl. 6 and 7) | Appropriate category selected by respondent |
| Household domestic assistance (not related to household head) | Household employment of a domestic worker | 1 = yes  0 = no |
| Main type of household toilet | 1. Private flush toilet  2. Shared flush toilet  3. Pit latrine  4. VIP Latrine  5. Bush/field  6. Bucket system  7. Chemical  8. Other | Appropriate category selected by respondent |
| Main source of household drinking water | 1. Piped to residence  2. Piped to yard  3. Public tap  4. Protected well  6. Traditional well  7. Borehole  8. River  9. Other (incl. 7 and 8) | Appropriate category selected by respondent |
| Fuel access for keeping household warm | 0. Nothing  1. Electricity  2. Petroleum gas  3. Kerosene  4. Charcoal  5. Wood | Appropriate category selected by respondent |
| Household reliance on food relief | Household reliance on food relief during the past 18 months | 1 = yes  0 = no |

Source: ZAMSTAR (2010).
