# Supplementary material for: Patient predictors of health-seeking behaviour for persons coughing for more than two weeks in high-burden tuberculosis communities: the case of the Western Cape, South Africa
Source: BMC Health Serv Res. 2019 Mar 13;19:160. doi: 10.1186/s12913-019-3992-6 (PMC6417175; doi:10.1186/s12913-019-3992-6)
Supplement: Supplementary file 2 — Correlation between components of asset index and asset index. Table showing correlation between components of asset index and asset index. (DOCX 14 kb) [file 12913_2019_3992_MOESM2_ESM.docx]

**Correlation between components of asset index and asset index**

| **Items** | **Coefficients (n= 30 017)** |
| --- | --- |
| Television | 0.568^***^ |
| Refrigeration | 0.471^***^ |
| Motor vehicle | 0.233^***^ |
| Mobile phone | 0.137^***^ |
| Electricity in the household | 0.758^***^ |
| House (single unit) | 0.401^***^ |
| House (multi-unit) | 0.280^***^ |
| Traditional dwelling | -0.067^***^ |
| Flat | 0.350^***^ |
| Room in backyard | -0.269^***^ |
| Worker hostel | 0.081^***^ |
| Other (incl. informal dwellings in backyard and not in backyard) | 0.027^***^ |
| Household employment of a domestic worker | 0.203^***^ |
| Private flush toilet | 0.666^***^ |
| Shared flush toilet | 0.240^***^ |
| Pit latrine | -0.126^***^ |
| VIP Latrine/chemical | -0.523^***^ |
| Bush/field | -0.249^***^ |
| Bucket system | 0.013^***^ |
| Piped to residence | 0.189^***^ |
| Piped to yard | 0.004^***^ |
| Public tap | -0.426^***^ |
| Protected well | 0.068^***^ |
| Other (incl. borehole, river, traditional well) | -0.333^***^ |
| Nothing | 0.027^***^ |
| Electricity | 0.225^***^ |
| Petroleum gas | 0.172^***^ |
| Kerosene | 0.070^***^ |
| Charcoal | -0.074^***^ |
| Wood | -0.167^***^ |
| Household reliance on food relief during the past 18 months | -0.089^***^ |

*** p<0.01, ** p<0.05, * p<0.1; Caravan (n=2) and other types of toilets (n=19) omitted due to collinearity. Source: Own calculations, ZAMSTAR (2010).
