# Supplementary material for: Patient predictors of health-seeking behaviour for persons coughing for more than two weeks in high-burden tuberculosis communities: the case of the Western Cape, South Africa
Source: BMC Health Serv Res. 2019 Mar 13;19:160. doi: 10.1186/s12913-019-3992-6 (PMC6417175; doi:10.1186/s12913-019-3992-6)
Supplement: Supplementary file 3 — TB-stigma indicator set. Table showing TB-stigma indicator set used in the SOCS study. (DOCX 13 kb) [file 12913_2019_3992_MOESM3_ESM.docx]

**TB-stigma indicator set**

| **Population group** | **Domain** | **Indicator coding ^a^** |
| --- | --- | --- |
| Household members of confirmed TB patient | Transmission myths (3 items) | 1 = agreed to any one of the three transmission myths  0 = disagreed with all |
| Household members of confirmed TB patient | Blame | 1 = agreed that TB is a punishment for being bad  0 = disagreed |
| Confirmed TB patient | Experience of social exclusion (4 items) | 1 = agreed to any one of the four examples of social exclusion  0 = disagreed with all |
| Confirmed TB patient | Experience of being made fun of (3 items) | 1 = agreed to any one of the three examples of being made fun of  0 = disagreed with all |
| Confirmed TB patient | Experience of health-setting stigma | 1 = yes  0 = no |
| Confirmed TB patient | Internalised stigma | 1 = yes  0 = no |
| Confirmed TB patient | Disclosure^b^ | 1= yes  0= no |

**^a^** Answers signaling stigma were coded ‘1’ while no stigma were coded ‘0’. ^b^ Disclosure domain excluded from stigma index. Source: SOCS of ZAMSTAR (2008/2009).
