# Supplementary material for: Patient predictors of health-seeking behaviour for persons coughing for more than two weeks in high-burden tuberculosis communities: the case of the Western Cape, South Africa
Source: BMC Health Serv Res. 2019 Mar 13;19:160. doi: 10.1186/s12913-019-3992-6 (PMC6417175; doi:10.1186/s12913-019-3992-6)
Supplement: Supplementary file 4 — Stigma level by community (a), age (b) and gender (c). Figures (a-c) showing mean stigma index levels (standardised using z-score) for each community (1–8), for each age category (< 18 years, 18–24 years, 25–45 years, 46–60 years, 61+ years) and for each gender (women and men). Source: own calculations, SOCS of ZAMSTAR (2008/2009). (DOCX 26 kb) [file 12913_2019_3992_MOESM4_ESM.docx]

(a)

(b)

(c)
